# SLC35E1 promotes keratinocyte proliferation in psoriasis by regulating zinc homeostasis

**DOI:** 10.1038/s41419-023-05874-1

**Published:** 2023-06-09

**Authors:** Tao Huang, Shijun Chen, Ke Ding, Baoqing Zheng, Weiqi Lv, Xiaobo Wang, Yadan Zhong, Hongxin Huang, Xin Zhang, Shufeng Ma, Bin Yang, Xiaohua Wang, Zhili Rong

**Affiliations:** 1 Cancer Research Institute, School of Basic Medical Sciences, State Key Laboratory of Organ Failure Research, National Clinical Research Center of Kidney Disease, Key Laboratory of Organ Failure Research (Ministry of Education), Southern Medical University, Guangzhou, 510515 China; 2 Dermatology Hospital, Southern Medical University, Guangzhou, 510091 China; 3 Affiliated Dongguan Hospital, Southern Medical University, (Dongguan People’s Hospital), Dongguan, 523058 China; 4 Department of Nephrology, Shenzhen Hospital, Southern Medical University, Shenzhen, 518110 China

**Keywords:** Psoriasis, Mechanisms of disease

## Abstract

Keratinocyte hyperproliferation is a key pathogenic factor in psoriasis. However, the mechanisms that regulate keratinocyte hyperproliferation in this condition remain unclear. Here, we found that SLC35E1 was highly expressed in keratinocytes of patients with psoriasis and that *Slc35e1*^*−*/*−*^ mice displayed a less severe imiquimod (IMQ)-induced psoriasis-like phenotype than their wild-type siblings. In addition, SLC35E1 deficiency inhibited keratinocyte proliferation in both mice and cultured cells. On a molecular level, SLC35E1 was found to regulate zinc ion concentrations and subcellular localization, while zinc ion chelation reversed the IMQ-induced psoriatic phenotype in *Slc35e1*^*−*/*−*^ mice. Meanwhile, epidermal zinc ion levels were decreased in patients with psoriasis and zinc ion supplementation alleviated the psoriatic phenotype in an IMQ-induced mouse model of psoriasis. Our results indicated that SLC35E1 can promote keratinocyte proliferation by regulating zinc ion homeostasis and zinc ion supplementation has potential as a therapy for psoriasis.

## Introduction

Psoriasis is a common chronic inflammatory cutaneous and systemic disease that affects ~2% of the world’s population [1, 2]. However, the precise mechanisms underlying the pathogenesis of psoriasis remain elusive. Psoriatic lesions are characterized by epidermal hyperplasia with abnormal keratinocyte proliferation and differentiation, as well as dramatic immune cell infiltration [2]. Epidermal hyperplasia in psoriasis is known to be the result of dysregulated crosstalk between epidermal keratinocytes and immune cells [2, 3].

Abnormal keratinocyte proliferation is a major histological manifestation of psoriasis and is induced by immune disorder [4]. Interleukin (IL)-17 and IL-22, two key psoriasis-related cytokines secreted by T-helper 17 cells, can promote keratinocyte proliferation [5]. Antimicrobial peptides, such as s100, LL37, and self-DNA, can also stimulate keratinocyte proliferation by activating the NF-κB and MAPK pathways [6].

Solute-linked carrier (SLC) transporters comprise a family of over 300 membrane-bound proteins that facilitate the transport of a wide range of substrates across biological membranes. SLCs play important roles in both physiological and pathophysiological processes [7, 8]. For example, mutant forms of SLC14A1, a urea transporter, have been implicated in bladder cancer [9], while SLC24A5 and SLC45A2 are involved in skin color determination [10, 11]. Several SLC subfamilies also participate in metal ion transport. Two members of the SLC11 subfamily, also known as natural resistance-associated macrophage proteins (NRAMPs) [12], function as proton-coupled metal ion influx transporters; 10 members of the SLC30 subfamily mediate Zn^2+^ efflux; and the SLC39 subfamily, with 14 members, functions in the influx of Zn^2+^, Fe^2+^, Cu^2+^, and Mn^2+^ [13, 14]. Ion channel-mediated ion flux influences skin barrier homeostasis by regulating keratinocyte proliferation and differentiation [15]. Clinical studies have suggested that hypocalcemia is a risk factor for psoriasis [16]. Zinc supplementation has been shown to ameliorate psoriasis both in vitro and in mice [17]. However, the role of metal ions in psoriasis remains unknown.

The physiological and pathological roles of SLC35E1, a member of the SLC35 family, are still unclear. Here, we investigated the contribution of SLC35E1 to keratinocyte hyperproliferation in psoriasis. We found that SLC35E1 is highly expressed in the suprabasal layer of psoriatic lesions. SLC35E1 deficiency inhibited keratinocyte proliferation in mice with imiquimod (IMQ)-induced psoriasis. Intriguingly, we found that zinc ion treatment suppressed the pathogenic manifestations in psoriatic mice. Our findings suggested that SLC35E1 may promote keratinocyte proliferation by regulating zinc levels, an effect that may be related to the pathogenesis of psoriasis.

## Materials and methods

### Human subjects

Psoriatic skin samples were obtained by punch biopsy from patients under local lidocaine anesthesia. Normal adult human skin specimens were acquired from healthy donors who were undergoing plastic surgery. The objectives, procedures, and potential risks were verbally explained to all the participants. Written informed consent (in Chinese) was obtained from all the participants before inclusion in the study.

### Animal model

*Slc35e1*-knockout (KO) mice, generated through the CRISPR-Cas9-mediated deletion of exon 2 to exon5 of the *Slc35e1* gene, were obtained from Cyagen (USA) and were maintained under specific pathogen-free conditions. To generate heterozygotes, *Slc35e1*^*−/−*^ mice were crossed with C57BL/6J mice obtained from the Animal Experiment Centre of Southern Medical University, GuangZhou, China. The heterozygotes were crossed to generate wild-type (WT) and *Slc35e1*^*−/−*^ mice. All animal experiments were performed in compliance with the Southern Medical University Animal Care and Use Committee guidelines. Mice were housed in individually ventilated cages at a maximum density of five mice per cage and kept on 12h-12 h light-dark cycle. Room temperature was maintained at 22 °C ± 1 °C with 30–70% humidity. Mice were fed ad libitum with rodent diet and water. None of the mice were involved in any previous procedures before the study. For the IMQ-induced model of psoriasis, sexual matched mice were mated at 7–9 weeks of age. The mice were administered a daily topical dose of 15 mg of IMQ cream (5%) per ear.

### Flow cytometry

Single-cell suspensions of the dorsal skin and ears were generated. The epidermis and dermis of the dorsal skin and ears of WT and *Slc35e1*^*−/−*^ mice were separated using 2.5 mg/ml dispase II (Sigma, USA) at 37 °C for 1 h, digested with collagenase type IV at 37 °C for respectively 1 and 2 h to generate single-cell suspensions, and stained with fluorophore-conjugated monoclonal antibodies against CD45 (103108, Biolegend), MCH-II (107622, Biolegend), F4/80 (17-4081-82, eBioscience), γδTCR (118131, Biolegend), Ly6G (127618, Biolegend), CD207 (144204, Biolegend), and CD11b (103208, Biolegend). After washing and resuspending, the cells were assayed with a BD LSRFORTESSA flow cytometer, and the data were analyzed using FlowJo software.

For zinc ion staining, mouse epidermal cells or Normal Human Epidermal Keratinocytes (NHEKs) were incubated with 10 µM FluoZin-3, AM (Invitrogen, USA) for 1 h at 37 °C in an incubator with 5% CO_2_. Then zinc ions were detected by flow cytometry.

### Histology

Paraffin-embedded skin specimens were sectioned, stained with hematoxylin and eosin (H&E), and visualized using a light microscope (Nikon, Eclipse 80i, Japan). Epidermal thickness (×100 magnification) was measured using ImageJ software using the following formula: thickness (µm) = area (µm^2^) / length (µm).

### RNA extraction and qPCR

Total RNA was extracted using TRIzol reagent (Yeasen Biotech, cat. 10606ES60) and reverse transcribed into cDNA using the Evo M-MLV RT Kit with gDNA Clean for qPCR II (Accurate Biotechnology cat. AG11711). Real-time quantitative PCR (qPCR) was conducted in a LightCycler 96 (Roche, Basel, Switzerland) using the SYBR Green Premix Pro Taq HS qPCR Kit (Accurate Biotechnology cat. AG11701). The relative expression levels of target genes were normalized to those of GAPDH and quantified using the 2^−ΔΔCt^ method. The sequences of the primers used for qPCR are presented in Supplementary Table 1.

### RNA-Seq

Total RNA was extracted from epidermal cells isolated from untreated and IMQ-treated WT and *Slc35e1*^*−/−*^ mice (three repetitions per group) using TRIzol Reagent (Invitrogen cat. 15596026). Total RNA was quantified and qualified using an Agilent 2100 Bioanalyzer (Agilent Technologies, Palo Alto, CA, USA), a NanoDrop (Thermo Fisher Scientific Inc.), and 1% agarose gel electrophoresis. Total RNA (1 μg) with a RIN > 6.5 was used for subsequent library preparation. Poly(A) mRNA was isolated using a Poly(A) mRNA Magnetic Isolation Module or a rRNA Removal Kit. mRNA fragmentation and priming were undertaken using First-Strand Synthesis Reaction Buffer and Random Primers. First-strand cDNA was synthesized using ProtoScript II Reverse Transcriptase and second-strand cDNA was synthesized using Second Strand Synthesis Enzyme Mix. The double-stranded cDNA was bead-purified and treated with End Prep Enzyme Mix to repair both ends and add a dA-tail in one reaction, followed by a T-A ligation to add adaptors to both ends. Size selection of adaptor-ligated DNA was then performed using beads and fragments of ~420 bp (with an insert size of ~300 bp) were recovered. Each sample was amplified by 13 cycles of PCR using P5 and P7 primers. Both primers carry sequences that can anneal to the flow cell for bridge PCR, while the P7 primer carries a six-base index allowing for multiplexing. The PCR products were cleaned using beads, validated using a Qsep100 (Bioptic, Taiwan, China), and quantified using a Qubit3.0 Fluorometer (Invitrogen, Carlsbad, CA, USA).

Libraries with different indices were multiplexed and loaded on an Illumina HiSeq instrument according to the manufacturer’s instructions (Illumina, San Diego, CA, USA). Sequencing was carried out using a 2× 150-bp paired-end configuration while image analysis and base calling were performed by the HiSeq Control Software (HCS) + OLB + GAPipeline-1.6 (Illumina) on the HiSeq instrument. For the psoriasis analysis, two RNA-Seq datasets (accession numbers: GSE54456 and GSE121212) were used, one for normal skin samples and one for psoriasis skin samples.

### RNA-Seq analysis

First, reference genome sequences and gene model annotation files of relative species were downloaded from the UCSC, NCBI, and ENSEMBL genome browsers. Then, Hisat2 (v2.0.1) was used to index reference genome sequences. Finally, clean data were aligned to the reference genomes using Hisat2 software (v2.0.1). Transcripts in FASTA format were initially converted from a known gff annotation file and correctly indexed. Then, with this file serving as a reference gene file, gene and isoform expression levels were estimated from the pair-end clean data using HTSeq (v0.6.1). The DESeq2 Bioconductor package, which uses the negative binomial distribution, was used for differential expression analysis. An adjusted *p*-value (*p*adj) of <0.05 was used to detect differentially expressed genes. GOSeq (v1.34.1) was used to identify enriched Gene Ontology (GO) terms among the differentially expressed genes at a padj of <0.05. TopGO was used to plot DAG. In-house scripts were used for KEGG pathway prediction for the differentially expressed genes.

### EdU cell proliferation assay

The thymidine analog 5-ethynyl-2′-deoxyuridine (EdU) has a terminal alkyne group replacing a methyl group at the 5-position of the pyrimidine ring and can be readily incorporated into DNA during synthesis [18]. EdU staining was used for detecting DNA synthesis in proliferating cells in vivo and in vitro. In vivo, EdU was intraperitoneally injected into mice 48 h before euthanasia. In vitro, keratinocytes were incubated with EdU for 2 h at 37 °C with CO_2_. Skin and keratinocytes were then stained with EdU using an EdU Cell Proliferation Kit (Beyotime,China).

### Immunofluorescence

After collection, mouse tissues were immediately embedded in OCT Compound (SAKURA cat. 4583), frozen at −80 °C, and cryosectioned into 7-µm slices using a freezing microtome (Leica, CM1900, Germany). The slices were blocked in 5% BSA for 45 min, incubated overnight with primary antibody at 4 °C, washed with PBS, and stained with fluorochrome-conjugated secondary antibody (mouse anti-rabbit antibody, Life Sciences, cat A11008; 1:500 dilution) for 1 h at room temperature in the dark.

For zinc ion staining, NHEKs were incubated with 10 µM FluoZin-3, AM (Invitrogen) for 1 h at 37 °C in an incubator with 5% CO_2_.

### Western blot

Total protein was extracted from skin using ice-cold RIPA lysis buffer, snap-freezing, and mechanical shearing. Protein supernatants were separated on 10% gradient polyacrylamide gels and then transferred to PVDF membranes (Millipore). After blocking with 5% non-fat dry milk for 1 h at room temperature, the membranes were incubated first with primary antibodies against SLC35E1 (Abcam, ab12150, 1:1000) or GAPDH (Proteintech, 60004-1-Ig, 1:5000) overnight at 4 °C, and then with secondary antibody for 1 h at room temperature. The signals were detected using Immobilon Western Chemiluminescent HRP Substrate (Millipore).

### Replicates and statistical analysis

Error bars in this study indicate means ± S.D. for qPCR. Unpaired, two-tailed Student’s *t*-tests were used for comparisons between two groups of at least three independent biological samples each. *P*-values < 0.05 were considered significant. All statistical analyzes were performed with GraphPad Prism 8 software.

## Results

### SLC35E1 was highly enriched in the suprabasal layer of skin lesions of patients with psoriasis

**Fig. 1:**
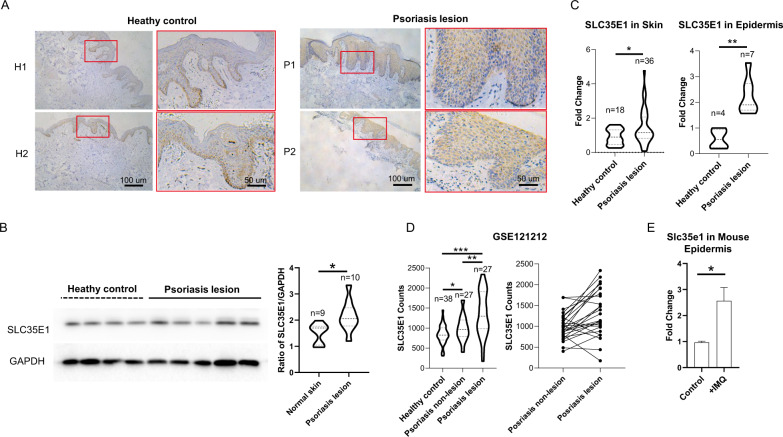
SLC35E1 is highly expressed in the suprabasal layer of psoriasis lesions. **A** Immunohistochemical staining for SLC35E1 in skin sections from healthy donors (*n* = 8) and patients with psoriasis (*n* = 7). **B** Western blot of SLC35E1 on whole-skin sections from 9 healthy donors and 10 patients with psoriasis. Representative images are shown on the left, and quantitative analysis on the right. **C** The mRNA levels of SLC35E1 in skin sections were assessed by RT-qPCR. Left: Whole skin from 18 healthy donors and 36 patients with psoriasis. Right: Epidermis from 4 healthy donors and 7 patients with psoriasis. **D** SLC35E1 is highly expressed in psoriatic lesions based on two RNA-Seq datasets from public databases (GSE54456 and GSE121212). In GSE54456, SLC35E1 counts in non-lesional psoriatic skin and lesional psoriatic skin from the same psoriasis patients in the GSE54456 dataset. **E** The mRNA level of *Slc35e1* was significantly higher in the epidermis of mouse psoriatic lesions (*n* = 3) and non-lesional skin (*n* = 3). Data are presented as means ± SEM; *P < 0.05, **P < 0.01, ***P < 0.001.

To gain insight into the expression profile of SLC35E1 in psoriatic lesions, we performed an immunohistochemical analysis of SLC35E1 in skin samples from patients with psoriasis and healthy controls. We observed that SLC35E1 was highly enriched in the suprabasal layer of psoriasis lesions (Fig. 1A), but was ubiquitously distributed in the basal layer of the epidermis in the controls. Consistent with this, immunoblotting and qPCR analysis showed that the protein and mRNA expression of SLC35E1 was significantly increased in psoriasis lesions compared with that in healthy skin (Fig. 1B, C and Fig. S1). Analysis of the GSE121212 dataset indicated that SLC35E1 expression was significantly higher in non-lesional psoriatic skin than in the skin of healthy controls, and was also higher in psoriatic lesions than in non-lesional psoriatic skin from the same psoriasis patients (Fig. 1D). These results suggested that abnormally elevated expression and ectopic tissue distribution of SLC35E1 protein in the suprabasal layer might be associated with the pathogenesis of psoriasis. Similarly, the mRNA level of SLC35E1 was significantly increased in the epidermis of mice in the IMQ model than in that of animals in the control (Fig. 1E).

### *Slc35e1* deficiency mitigated the IMQ-induced psoriasis-like phenotype

**Fig. 2:**
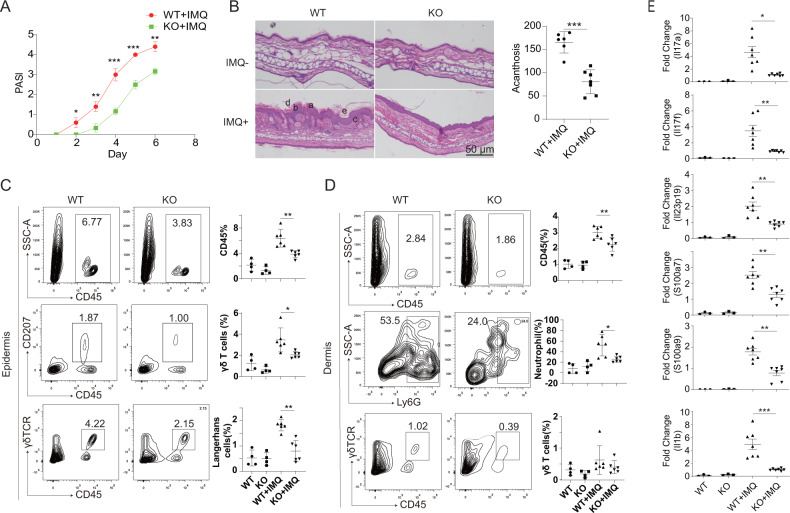
*Slc35e1* deficiency ameliorates the psoriasis-like phenotype in imiquimod (IMQ)-treated mice. Mice were subjected to a daily dose of 15 mg of IMQ cream per ear for 6 consecutive days. **A** The Psoriasis Area and Severity Index (PASI) score was recorded every day for both wild-type (WT) and *Slc35e1*-knockout (KO) mice (*n* = 3). **B** Hematoxylin and eosin (H&E) staining of non-lesional and lesional skin of WT (*n* = 6) and *Slc35e1*-KO (*n* = 6) mice with IMQ-induced psoriasis. (a, acanthosis; b, increased proliferative basal layer epidermal keratinocytes; c, dermal cell infiltrates; d, hyperkeratosis; e, parakeratosis. Left: Representative images. Right: Quantitative analysis of acanthosis in *Slc35e1*-KO and WT skin. **C**, **D** Infiltration of CD45+ leukocytes in the dermis and epidermis of WT (*n* = 4), KO (*n* = 4), WT + IMQ (*n* = 6), and KO + IMQ (*n* = 6) mice. **C** Epidermal (γδ T cells and Langerhans cells) and **D** dermal (neutrophils and γδ T cells) infiltration was detected by flow cytometry after treatment with dispase II. Left: Representative data. Right: Quantitative analysis. **E** The mRNA level of psoriatic inflammatory factors in the skin of WT (*n* = 3), KO (*n* = 3), WT + IMQ (*n* = 7), and KO + IMQ (*n* = 7) mice. Data are presented as means ± SEM; *P < 0.05, **P < 0.01, ***P < 0.001.

To investigate the function of SLC35E1 in the pathogenesis of psoriasis, we established a mouse model of psoriasis in *Slc35e1*^*−/−*^ mice *via* the topical application of IMQ, with their WT siblings serving as controls. Interestingly, we found that *Slc35e1*^*−/−*^ mice had significantly lower body weight (Fig. S2). In the IMQ mouse model of psoriasis, the Psoriasis Area and Severity Index (PASI) score was recorded to evaluate disease severity, with the result showing that the PASI score was significantly lower in *Slc35e1*^*−/−*^ mice than in the WT controls (Fig. 2A). Furthermore, histological analysis of the skin of IMQ-treated control mice showed the presence of notable psoriasis-like skin lesions, including epidermal hyperplasia (acanthosis), parakeratosis in the epidermis, and dermal hyperkeratosis (Fig. 2B). In contrast, the psoriasis-like skin lesions were significantly alleviated in *Slc35e1*^*−/−*^ mice. As psoriasis is characterized by the infiltration of inflammatory cells and excess expression of inflammatory cytokines, we next undertook a flow cytometry-based analysis of inflammatory cells (CD45+ cells, Langerhans cells, gamma delta T cells, macrophages, and neutrophils) in the epidermis and dermis. We found that the percentages of the tested inflammatory cells were significantly lower in the skin lesions of *Slc35e1-*KO mice than in those of the WT controls (Fig. 2C, D). Similarly, the qPCR analysis indicated that the expression of psoriasis-related cytokines, such as IL-17a and IL-17f, was significantly lower in *Slc35e1*^*−/−*^ mice than in control animals (Fig. 2E and Fig. S3). These results suggested that knocking out *Slc35e1* could inhibit the development of psoriasis.

### SLC35E1 deficiency inhibited keratinocyte proliferation

**Fig. 3:**
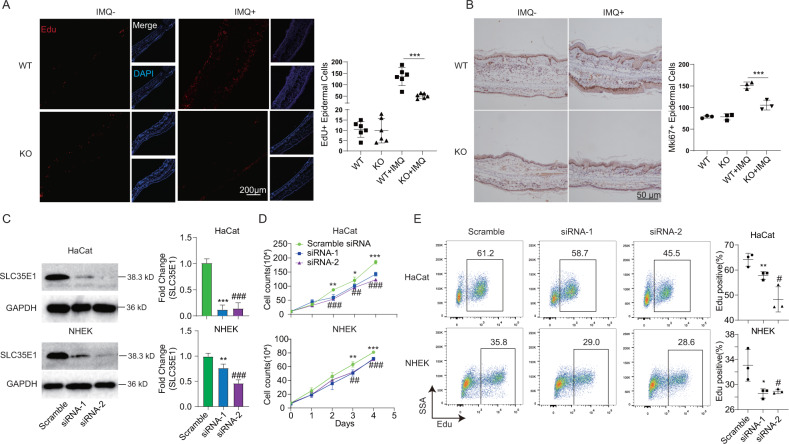
SLC35E1 deficiency inhibits the proliferation of keratinocytes. **A**, **B** EdU and Ki67 staining in psoriasis-like lesions and non-lesional skin of mice with imiquimod (IMQ)-induced psoriasis. **A** EdU staining. During the IMQ modeling, EdU was injected into WT(*n* = 3), KO (*n* = 3), WT + IMQ (*n* = 6), and KO + IMQ (*n* = 6) mice 48 h before euthanasia. Left: Representative images. Right: Quantitative analysis. **B** Immunohistochemistry using a rabbit polyclonal antibody directed against Ki67 in WT (*n* = 3), KO (*n* = 3), WT + IMQ (*n* = 6), and KO + IMQ (*n* = 6) mice. **C–E** The knockdown of SLC35E1 in keratinocytes inhibits their proliferation. **C** SLC35E1 was knockdown by RNAi. Two independent siRNAs targeting SLC35E1 were transfected into HaCats and Normal Human Epidermal Keratinocytes (NHEKs) and the expression of SLC35E1 was detected by western blot (left) and RT-qPCR (right). **D** Proliferation curve of keratinocytes after SLC35E1 knockdown. **E** Quantitative analysis of keratinocyte proliferation by flow cytometry. The cells were incubated with EdU for 2 h and then EdU-positive cells were detected by flow cytometry. The data are representative of three independent experiments. Data are presented as means ± SEM; *^/#^*P* < 0.05, ^**/##^*P* < 0.01, ^***/###^*P* < 0.001; * represents siRNA-1 *vs*. sramble siRNA and ^#^ represents siRNA-2 *vs*. sramble siRNA.

The immunohistochemistry results showed that SLC35E1 was highly expressed in the suprabasal layer of the psoriatic epidermis, but only weakly expressed in the basal layer (Fig. 1A). The hyperproliferation of epidermal keratinocytes is an important feature of psoriasis. Hyperproliferation in psoriatic epidermis is not restricted to the basal epidermal layer, where keratinocyte stem cells reside, but may also involve suprabasal cells, suggesting that SLC35E1 may regulate keratinocyte proliferation. To test this possibility, we quantified the number of proliferating cells in skin lesions of IMQ-treated WT and *Slc35e1*^*−/−*^ mice using EdU staining. We found that the number of EdU-positive (proliferating) cells was significantly lower in the lesional epidermis of *Slc35e1* KO mice than in that of the WT controls (Fig. 3A). Furthermore, there were significantly fewer Ki67-positive keratinocytes in the skin lesions of KO mice than in those of WT animals as determined by immunohistochemistry (Fig. 3B). In contrast, no significant difference in staining intensity for markers of differentiation (loricrin and filaggrin) and apoptosis (cleaved caspase-3) in skin lesions were observed between *Slc35e1*^*−/−*^ and control mice (Fig. S4A). We further assessed whether SLC35E1 depletion modulated keratinocyte proliferation in NHEKs and HaCats by silencing SLC35E1 with two independent siRNAs (Fig. 3C and Fig. S5). The results showed that, compared with the controls, the total number of cells and the number of proliferating cells were both significantly decreased after siRNA-mediated silencing of SLC35E1 (Fig. 3D, E). In contrast, no difference in the number of apoptotic cells was detected as determined by flow cytometric analysis (Fig. S4B). Together, these findings suggested that SLC35E1 deficiency inhibits the proliferation of keratinocytes, but not their differentiation or apoptosis, both in vivo and in vitro.

### SLC35E1 regulated zinc ion concentration and localization in keratinocytes

**Fig. 4:**
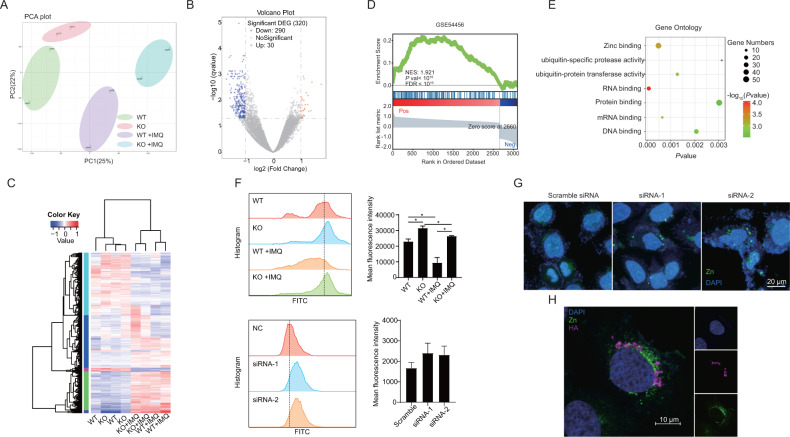
SLC35E1 regulates zinc ion concentrations and localization in keratinocytes. **A**–**E** RNA sequencing of the epidermis of wild-type (WT) (*n* = 2), knockout (KO) (*n* = 2), WT+ imiquimod (IMQ) (*n* = 2), and KO + IMQ (*n* = 2) mice. **A** Principal component analysis of the transcriptome of epidermal cells from the WT group (green dots), KO group (red dots), WT + IMQ group (violet dots), and KO + IMQ group (blue dots). **B** Volcano plots showing the log2 fold-change (FC) (vertical gray lines) and the nominal *q*-value (gray horizontal line) for all the transcripts detected by the RNA-Seq in the WT + IMQ *versus* KO + IMQ group comparison. Gray dots indicate transcripts not significantly changed, blue dots represent transcripts with FC < 2, and red dots represent transcripts with FC > 2. **C** Heatmap of differentially expressed genes based on the RNA-Seq data. **D** Gene Set Enrichment Analysis (GSEA) of differentially expressed genes in the KO + IMQ group relative to the WT + IMQ group in human psoriatic skin (GSE54456). **E** Gene Ontology (GO) enrichment analysis of differentially expressed genes in the KO + IMQ group *versus* the WT + IMQ group. The size indicates the gene numbers and the color corresponds to the *P*-value. **F** SLC35E1 deficiency increases the zinc ion concentration in keratinocytes. Top: The zinc ion concentration was detected by flow cytometry after the separation of epidermal cells from WT (*n* = 3), KO (*n* = 3), WT + IMQ (*n* = 3), a*n*d KO + IMQ (*n* = 3) mice. Bottom: The zinc ion concentration was detected by flow cytometry 48 h after siRNA transfection into HaCat and Normal Human Epidermal Keratinocytes (NHEKs). **G** Zinc ion localization was changed after the knockdown of SLC35E1. Zinc ions were fluorescently stained after the transfection of siRNAs targeting SLC35E1 into NHEKs. **H** SLC35E1 partially co-localizes with zinc ions around the nucleus. NHEKs were transfected with the HA-tagged SLC35E1 eukaryotic expression vector followed by co-staining for the HA tag and zinc ions. Data are presented as means ± SEM; *P < 0.05, **P < 0.01, ***P < 0.001.

To identify which genes are associated with the function of SLC35E1, we next performed RNA-Seq for epidermal cells derived from *Slc35e1*^*−/−*^ and WT mice treated or not with IMQ, and found a notable difference in gene expression patterns between the two groups of mice (Fig. 4A, C), with 320 genes in total being identified as differentially expressed (jlog2FCj > 1 and *P* < 0.05) (Fig. 4B). However, the difference between untreated KO and control mice was less pronounced. Interestingly, the GSE analysis indicated that the differentially expressed genes identified between psoriatic lesions of *Slc35e1*^*−/−*^ and control mice showed significant overlap with those identified between the skin lesions of 92 human psoriatic skin samples and 82 normal skin samples (Fig. 4D). Furthermore, we found that SLC35E1 may regulate Notch and Wnt signaling pathways (Fig. S6). Studies have shown that Notch and Wnt signaling in epidermal keratinocytes is dysregulated in psoriasis. In addition, GO term enrichment analysis indicated that zinc ion binding was one of the most upregulated pathways in the epidermis of *Slc35e1* KO mice (Fig. 4E), suggesting that SLC35E1 might regulate zinc ion homeostasis. It has been reported that zinc levels are decreased in the serum of psoriatic patients [19]. In addition, the skin lesions of psoriatic patients, such as acanthosis and parakeratosis, are histologically similar to those encountered in zinc-deficient animals [20], suggesting that zinc ion homeostasis may be associated with psoriasis. To determine whether SLC35E1 is involved in the regulation of zinc ion homeostasis, we measured zinc ion concentrations in epidermal cells of WT and KO mice by flow cytometry. As expected, zinc ion concentrations were higher in epidermal cells of *Slc35e1* KO mice than in those of the controls. In vitro, zinc ion concentrations in keratinocytes were also increased when SLC35E1 was knocked down by RNAi (Fig. 4F). Combined, the in vivo and in vitro results suggested that SLC35E1 may mediate zinc ion homeostasis in the epidermis. Our findings also showed that the concentration of zinc ions was decreased in the epidermis of WT mice following IMQ treatment. Next, we performed zinc ion staining in NHEKs after the knockdown of SLC35E1 and found that, compared with the controls, zinc ion staining was brighter and more aggregated in the cytoplasm after SLC35E1 knockdown (Fig. 4G). We also assessed whether SLC35E1 co-localizes with zinc ions by co-staining for zinc ions and HA-tag-SLC35E1 in NHEKs transfected with a construct expressing HA-tagged SLC35E1. The results demonstrated that SLC35E1 partially co-localizes with zinc ions in the cytoplasm surrounding the nucleus (Fig. 4H).

### Epidermal zinc ion concentration was decreased in psoriasis

**Fig. 5:**
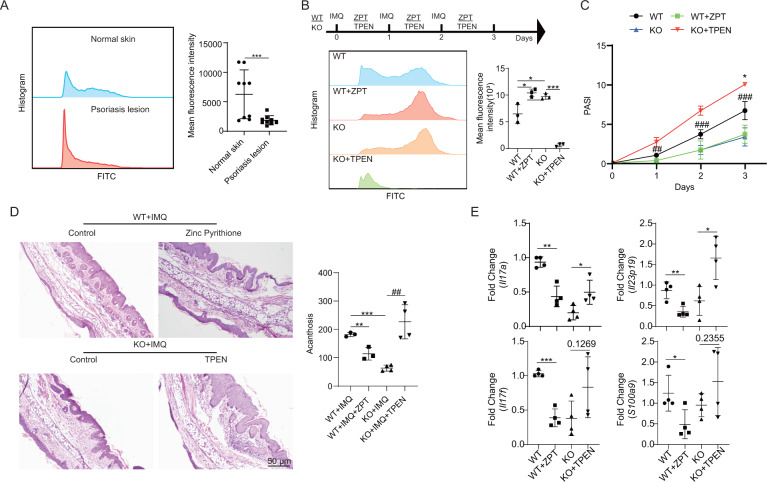
Zinc supplementation alleviates the psoriasis-like phenotype. **A** Quantitative analysis of epidermal zinc ion levels in healthy skin and psoriatic lesions. The epidermal zinc ion concentration in healthy skin (*n* = 9) and psoriatic lesions (*n* = 9) was detected by flow cytometry. **B** Quantitative analysis of epidermal zinc ion concentrations after TPEN and/or zinc pyrithione (ZPT) treatment. Wild-type (WT) mice (*n* = 3) were administered ZPT after being treated with IMQ for 12 h every day, while *Slc35e1*-knockout (KO) mice (*n* = 3) were treated with TPEN after being treated with IMQ for 12 h every day. ZPT was administered as a zinc ion supplement. TPEN, an intracellular membrane-permeable ion chelator, was used to lower zinc ion concentrations. The epidermal zinc ion concentration in each group (*n* = 3) was detected by flow cytometry. **C** The Psoriasis Area and Severity Index (PASI) score for WT + IMQ (*n* = 4), KO + IMQ (*n* = 4), WT + IMQ + ZPT (*n* = 4), and KO + IMQ + TPEN (*n* = 4) mice. **D** H&E staining. **E** The mRNA level of psoriasis-related inflammatory factors. Data are presented as means ± SEM; *P < 0.05, **P < 0.01, ***P < 0.001.

Here, our data showed that the epidermal zinc ion concentration was decreased in mice with IMQ-induced psoriasis. Similarly, a meta-analysis [19] reported that zinc ion plasma levels are low in patients with psoriasis. To further establish the levels of zinc ions in psoriatic lesions, we next measured the zinc ion concentration in the epidermis of healthy skin and psoriasis lesions using flow cytometry. The results showed that the epidermal zinc ion concentration was decreased in psoriatic lesions than in healthy skin (Fig. 5A).

### Zinc ion supplementation ameliorated psoriasis-like phenotypes

As shown in Fig. 4A, we observed that zinc ion concentrations were significantly lower in IMQ-treated WT mice, which showed a typical psoriasis phenotype, than in IMQ-treated *Slc35e1*^*−/−*^ mice, which displayed a less severe psoriasis-like phenotype. This suggested that a reduction in zinc ion concentrations may promote psoriasis and vice versa. To test whether increasing zinc ion concentrations could ameliorate the psoriatic phenotype, IMQ-treated WT mice were topically administered a zinc ion supplement, zinc pyrithione, a complex used in a variety of hair and skin care products. Similarly, to test whether decreasing zinc ion concentrations could worsen the psoriatic phenotype, IMQ-treated *Slc35e1*^*−/−*^ mice were topically administered an intracellular membrane-permeable zinc ion chelator, TPEN (N,N,N′,N′-tetrakis(2-pyridinylmethyl)-1,2-ethanediamine). As expected, zinc ion concentrations in epidermal cells were significantly increased in IMQ-treated WT mice administered zinc pyrithione and significantly decreased in IMQ-treated *Slc35e1*^*−/−*^ mice following TPEN application (Fig. 5B).

We also observed that a negative correlation existed between the PASI score and the zinc ion concentration, that is, the PASI score was decreased in IMQ-treated WT mice administered zinc pyrithione, but was increased in IMQ-treated *Slc35e1*^*−/−*^ mice after TPEN treatment (Fig. 5C). In line with these findings, the histopathological features, including acanthosis and inflammatory cell infiltration, were improved in IMQ-treated WT mice after zinc pyrithione application, whereas the opposite was seen in IMQ-treated *Slc35e1* KO mice after TPEN administration (Fig. 5D, E). To test whether zinc ions play a role in the regulation of keratinocyte proliferation, we treated NHEKs with zinc pyrithione to increase the concentration of zinc ions and then quantified the cell numbers. We found that the proliferative ability of NHEKs was decreased following zinc ion supplementation (Fig. S7).

## Discussion

Our study showed that SLC35E1, a previously uncharacterized SLC transporter, regulates the zinc ion-mediated proliferation of psoriatic keratinocytes. SLC transporters play important roles in numerous physiological and pathological processes [8]; however, the functions of many, including SLC35E1, remain to be fully characterized. Here, we showed that *Slc35e1*^*−/−*^ mice display a less severe psoriasis-like phenotype than their WT counterparts. We further demonstrated that SLC35E1 regulates keratinocyte proliferation both in vivo and in vitro, while our RNA-Seq results for *Slc35e1*^*−/−*^ mice suggested that *Slc35e1* may be involved in the regulation of zinc ion homeostasis. A clinical trial reported that zinc ions may alleviate psoriasis [21]. Here, we showed that SLC35E1 regulates zinc ion-mediated keratinocyte proliferation.

Keratinocyte hyperproliferation is a major pathogenic factor in psoriasis. Moreover, it has been suggested that this hyperproliferation is not restricted to the basal epidermal layer, which contains keratinocyte stem cells, but may also involve suprabasal cells [22, 23]. We found that SLC35E1 protein is highly expressed in the suprabasal layer of psoriatic lesions, suggesting that SLC35E1 may regulate the proliferation of psoriatic keratinocytes. Our findings further demonstrated that SLC35E1 regulates the proliferation of keratinocytes in vivo and in vitro. In addition to the association with psoriasis, we observed that *Slc35e1*^*−/−*^ mice have significantly lower body weight than their WT siblings (Fig. S1), suggesting that SLC35E1 may also play a role in development.

Studies have shown that serum zinc ion concentrations are lower in patients with psoriasis than in healthy controls [24–26]. Moreover, one clinical study showed that zinc ion supplementation can alleviate the symptoms of psoriasis [21]. Combined, these studies implied that low zinc ion concentrations may be a key factor in the pathogenesis of psoriasis; however, the underlying mechanism has remained unclear. Here, we showed that SLC35E1 regulates zinc ion homeostasis in keratinocytes. To assess the contributions of zinc ions to psoriasis pathology, we applied zinc pyrithione and TPEN, agents with opposite functions, to treat IMQ-induced psoriasis-like skin. The results show that zinc ion supplementation can alleviate the symptoms of psoriasis. In IMQ mouse model of psoriasis, the phenotype of SLC35E1 was rescued by reducing the concentration of zinc ions. It remains that SLC35E1 reduces zinc ion concentration to promote psoriasis in keratinocytes.

The homeostasis of zinc ions is mainly regulated by zinc ion transporters. Zinc transporters are divided into two major families, SLC39s/ZIPs and SLC30s/ZnTs, which transport zinc in opposite directions through cellular and intracellular membranes [27]. Here, our staining results showed that SLC35E1 and zinc ions partially co-localize in keratinocytes, suggesting that SLC35E1 may act as a zinc ion transporter. However, we were not able to provide more evidences for this in our study.

In summary, we demonstrated that a previously uncharacterized SLC transporter, SLC35E1, is associated with the hyperproliferation of psoriatic keratinocytes. We further showed that the role of SLC35E1 in psoriasis is to regulate zinc ion homeostasis. In psoriasis, epidermal zinc ion concentrations are significantly reduced, and zinc ion supplementation may represent a means of delivering adjuvant therapy for this condition.

## Supplementary information

Supplementary Figures

aj-checklist

## Data Availability

The RNA-Seq data have been deposited in the NCBI BioProject database under the accession number PRJNA860003.
