# Supplementary material for: SLC35E1 promotes keratinocyte proliferation in psoriasis by regulating zinc homeostasis
Source: Cell Death Dis. 2023 Jun 9;14(6):354. doi: 10.1038/s41419-023-05874-1 (PMC10256760; doi:10.1038/s41419-023-05874-1)
Supplement: Supplementary file 1 — Supplementary Figures [file 41419_2023_5874_MOESM1_ESM.pdf]

Supplemental Material

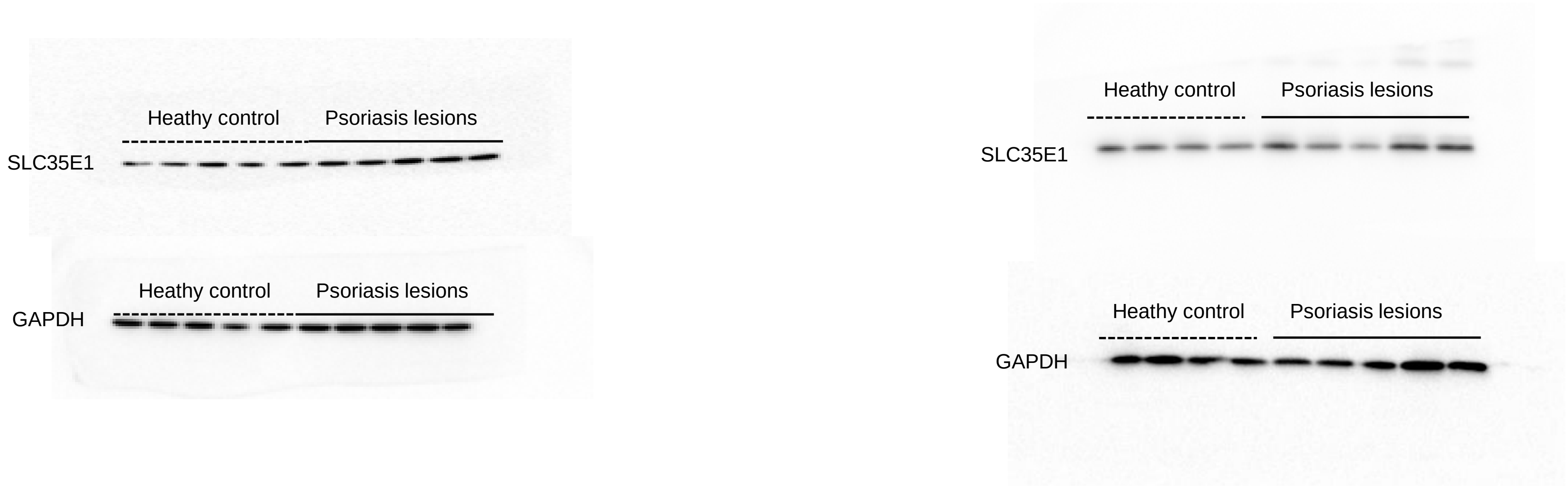

Figure S1. Western blot of SLC35E1 on whole-skin sections from 9 healthy donors and 10 patients with psoriasis.

	$Slc35e1^{+/+}$	$Slc35e1^{+/-}$	$Slc35e1^{-/-}$
Counts	22	41	24
Ratio	25%	47%	28%

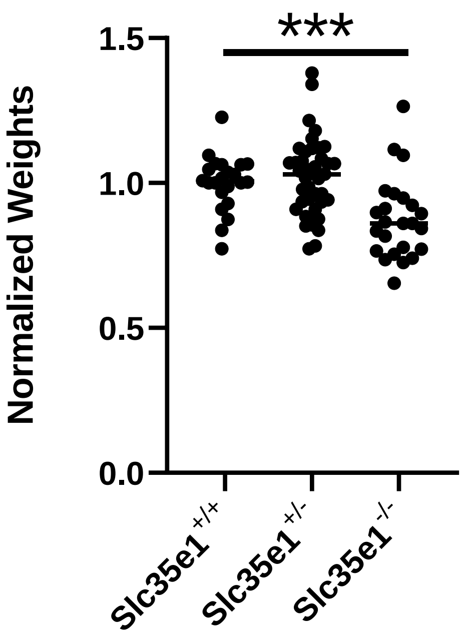

Figure S2. Slc35e1-KO mice had lower body weight. Mice were weighed at 8 weeks prior to IMQ-induced psoriasis. We performed breeding with heterozygotes for SLC35E1.

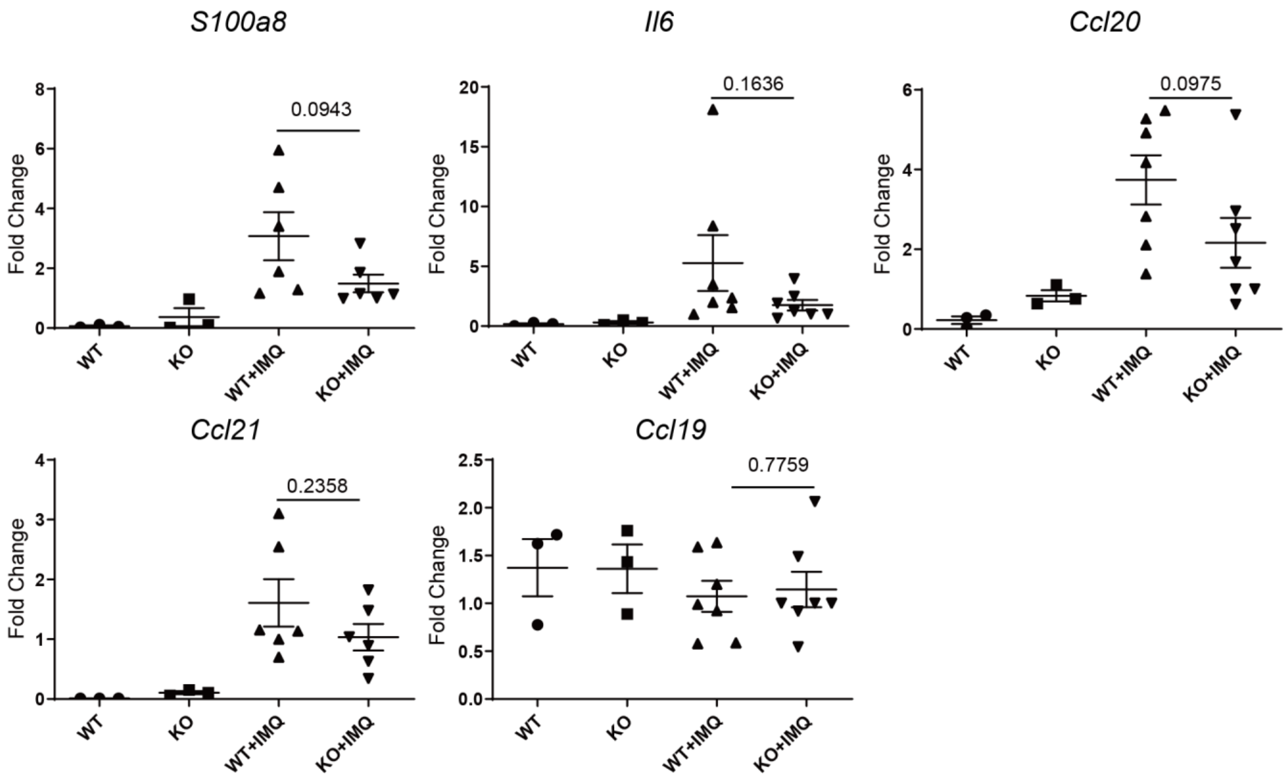

Figure S3. The mRNA level of psoriasis-related inflammatory factors in mouse skin.

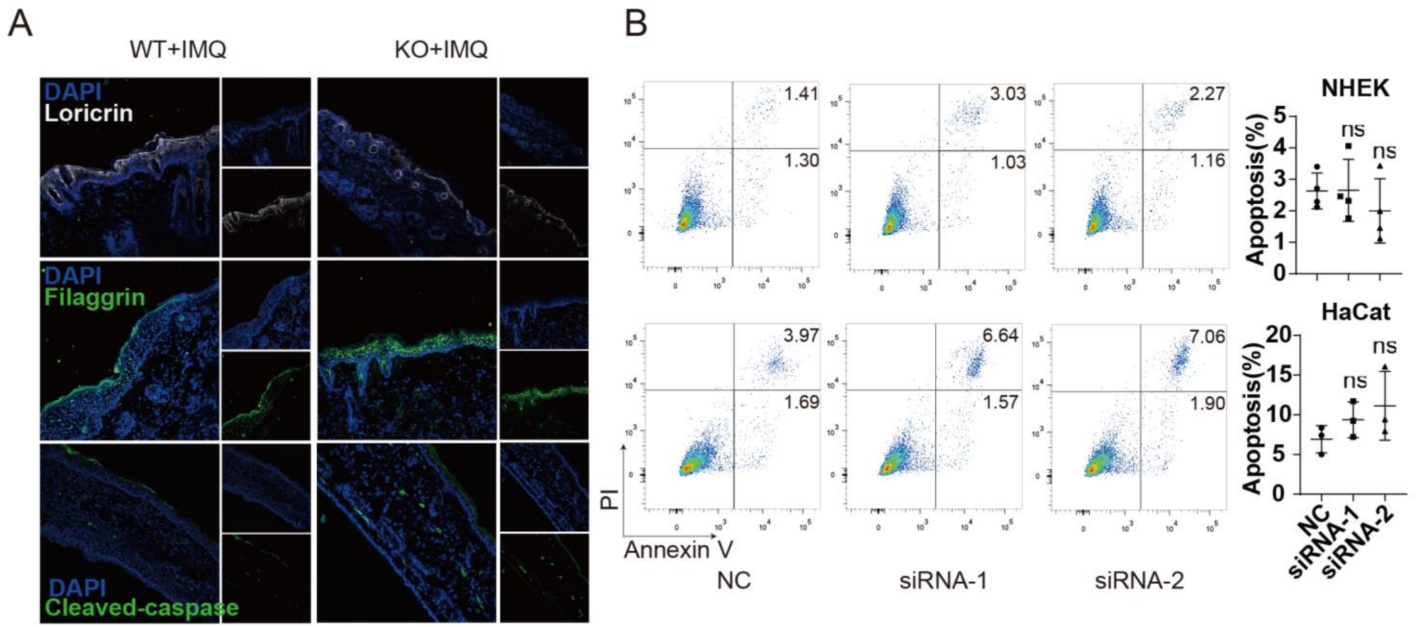

Figure S4. SLC35E1 did not regulate keratinocyte differentiation and apoptosis. (A) Staining for markers of differentiation (Loricrin and Filaggrin) and apoptosis (Cleaved-caspase). In IMQ mouse model of psoriasis, the differentiation markers Loricrin and Filaggrin and the apoptosis marker Cleaved-caspase were stained by immunofluorescence staining; (B) Knocking down SLC35E1 did not regulate apoptosis in human keratinocytes. After knocking down SLC35E1, apoptosis was detected by Annexin V/PI staining.

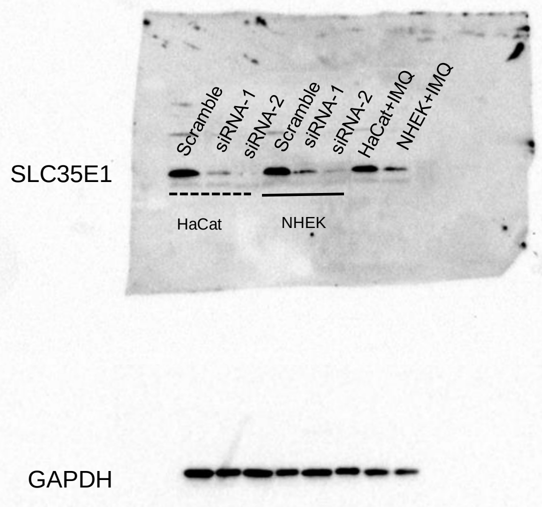

Figure S5. Western blot of SLC35E1. Two independent siRNAs targeting SLC35E1 were transfected into HaCats and Normal Human Epidermal Keratinocytes (NHEKs) and the expression of SLC35E1 was detected by western blot.

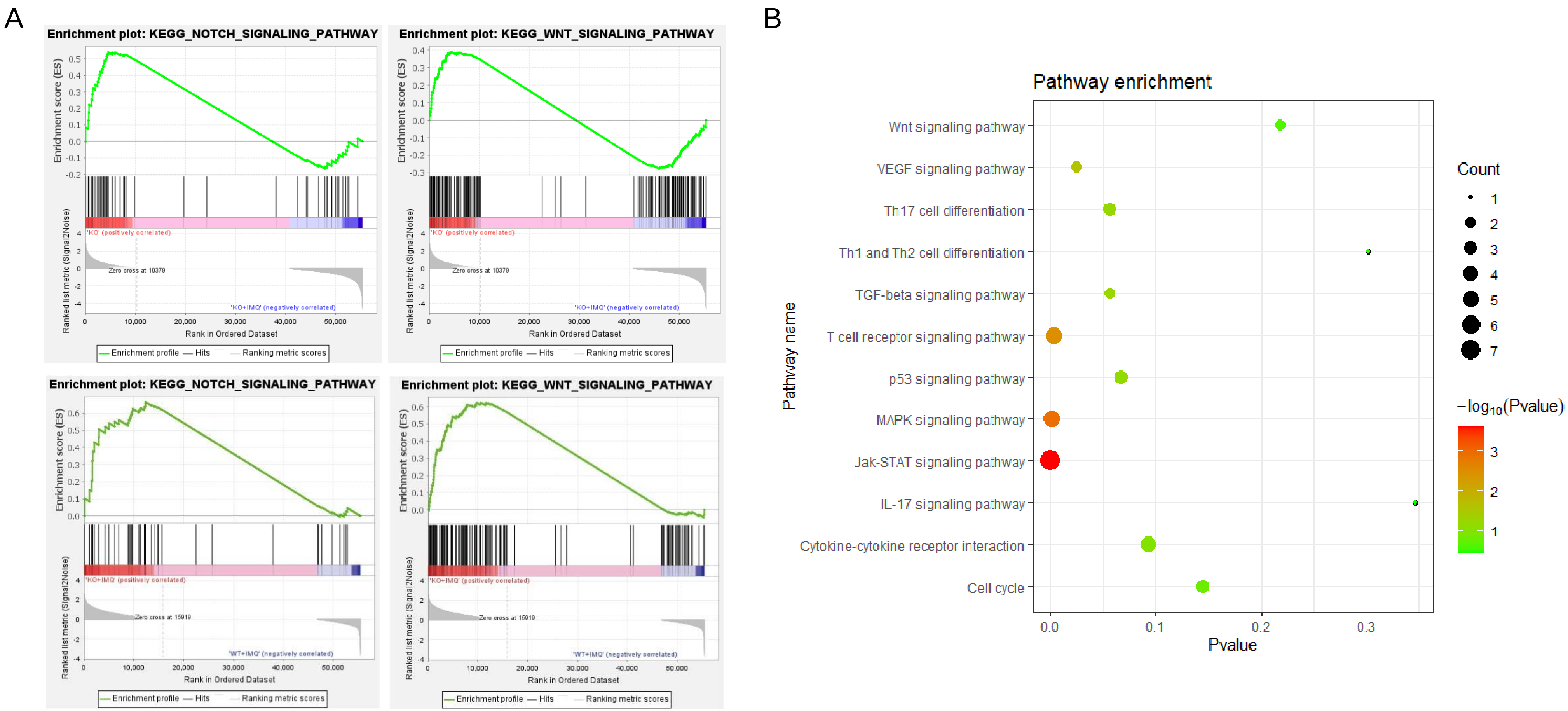

Figure S6. SLC35E1 deficiency affects psoriasis signaling pathways. (A) GSEA of genes in SLC35E1-KO to WT epidermis with Wnt and Nocth target signatures. (B) KEGG of DEGs in SLC35E1-KO relative to WT epidermis.

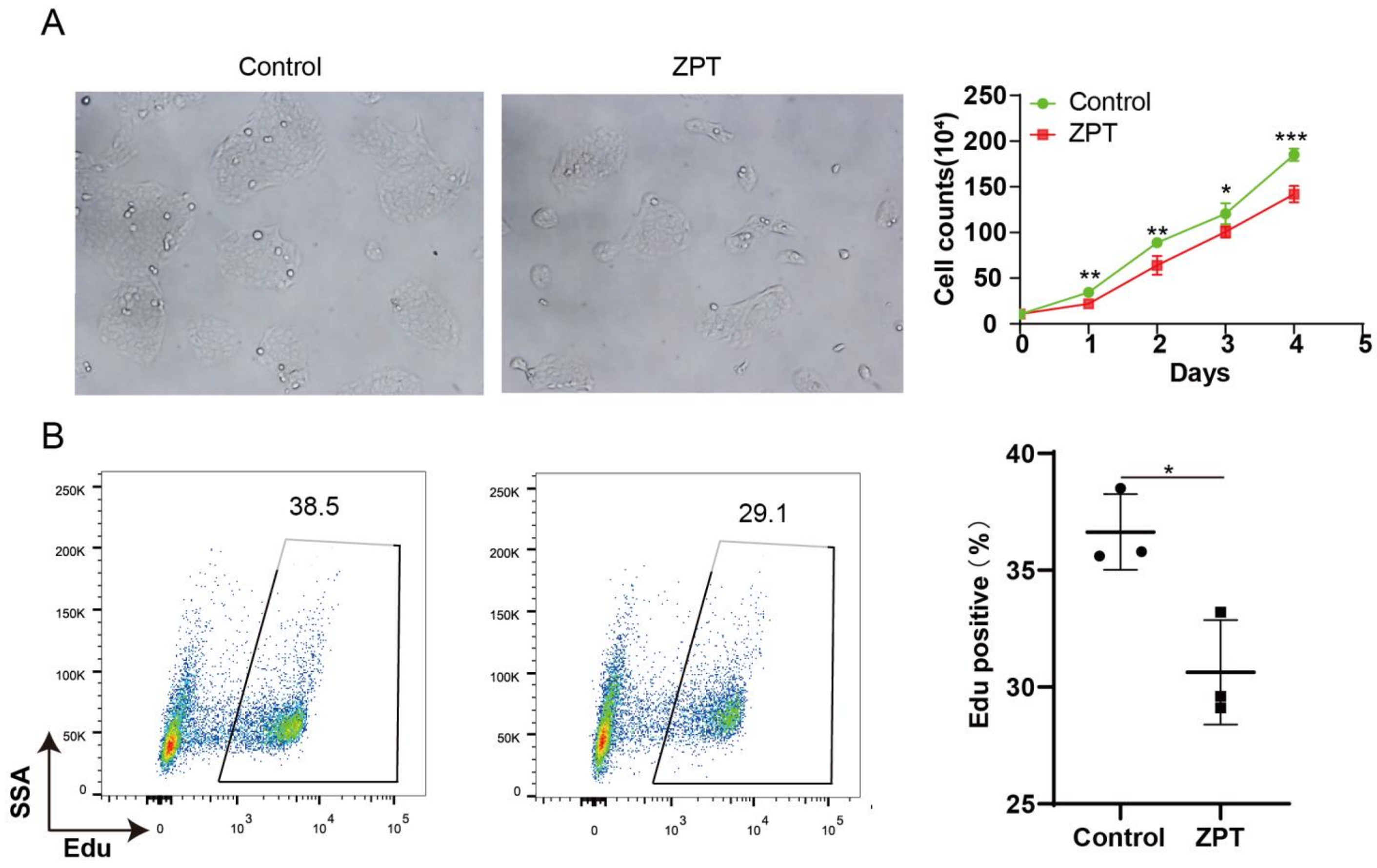

Figure S7. Zinc ion supplementation inhibits keratinocyte proliferation in vitro. (A) NHEK cell numbers were decreased with ZPT. ZPT is used to supplement zinc ions. Left: Photograph of NHEK cells after treat with ZPT four days; Right: Statistics of daily cell counts. (B) EdU positive NHEK cells were decreased with ZPT. Left: Representative images. Right: Quantitative analysis.

Table 1: Primers

Gene	Sequence
Gapdh-F	AACTTTGGCATTGTGGAAGG
Gapdh-R	ACACATTGGGGGTAGGAACA
Il-1b-F	GAAATGCCACCTTTTGACAGTG
Il-1b -R	TGGATGCTCTCATCAGGACAG
Ccl19-F	GCC TCA GAT TAT CTG CCA T
Ccl19-R	AGA CAC AGG GCT CCT TCT GGT
Ccl20-F	GCCTCTCGTACATACAGACGC
Ccl20-R	CCAGTTCTGCTTTGGATCAGC
Ccl21-F	GAA AAT TCC CTA CAG TAT TGT CCG AG
Ccl21-R	GAC TTA GAG GTT CCC CGG TTC
Il17a-F	CTCCAGAAGGCCCTCAGACTAC
Il17a-R	GGGTCTTCATTGCGGTGG
Il17f-F	CCCATGGGATTACAACATCACTC
Il17f-R	CACTGGGCCTCAGCGATC
Il23p19-F	AATGTGCCCCGTATCCAGTG
Il23p19-R	CAAGCAGAACTGGCTGTTGTC
Il6-F	CACTTCACAAGTCGGAGGCTTA
Il6-R	GCAAGTGCATCATCGTTGTTC
Tnfa-F	CAGGCGGTGCCTATGTCTC
Tnfa-R	CGATCACCCCGAAGTTCAGTAG
S100a7-F	TGCTCTTGGATAGTGTGCCTC
S100a7-R	GCTCTGTGATGTAGTATGGCTG
S100a8-F	AGTGTCCTCAGTTTGTGCAG
S100a8-R	ACTCCTTGTGGCTGTCTTTG
S100a9-F	ATACTCTAGGAAGGAAGGACACC
S100a9-R	TCCATGATGTCATTTATGAGGGC
