# Supplementary material for: SLC35E1 promotes keratinocyte proliferation in psoriasis by regulating zinc homeostasis
Source: Cell Death Dis. 2023 Jun 9;14(6):354. doi: 10.1038/s41419-023-05874-1 (PMC10256760; doi:10.1038/s41419-023-05874-1)
Supplement: Supplementary file 2 — aj-checklist [file 41419_2023_5874_MOESM2_ESM.pdf]

SPRINGER NATURE

Corresponding Author Name: zhili rong
Manuscript Number: CDDIS-23-0435

Reporting Summary

Springer Nature wishes to improve the reproducibility of the work that we publish. This checklist is used to ensure good reporting standards and to improve the reproducibility. Please respond completely to all questions relevant to your manuscript. For more information, please read the journal's Guide to Authors.

☑ **Check here to confirm that the following information is available in the Material & Methods section:**

- **The exact sample size (*n*)** for each experimental group/condition, given as a number, not a range
- **A description of the sample collection** allowing the reader to understand whether the samples represent technical or biological replicates (including how many animals, litters, culture, etc.)
- **A statement of how many times the experiment shown was replicated in the laboratory**
- **Definitions of statistical methods and measures**: For small sample sizes (n<5) descriptive statistics are not appropriate, instead plot individual data points
  - Very common tests, such as *t*-test, simple χ^2 tests, Wilcoxon and Mann-Whitney tests, can be unambiguously identified by name only, but more complex techniques should be described in the methods section
  - Are tests one-sided or two-sided?
  - Are there adjustments for multiple comparisons?
  - **Statistical test results**, e.g., ***P* values**
  - Definition of **'center values'** as **median or mean**;
  - Definition of **error bars as s.d. or s.e.m. or c.i.**

Please ensure that the answers to the following questions are reported in the manuscript itself. We encourage you to include a specific subsection in the methods section for statistics, reagents and animal models. Below, provide the page number or section and paragraph number.

Statistics and general methods

Question	Reported in section/paragraph or page #
1. How was the sample size chosen to ensure adequate power to detect a pre-specified effect size? (Give section/paragraph or page #)	No·pre-specified study in this study.
For animal studies, include a statement about sample size estimate even if no statistical methods were used.	The sample size was commonly determined according to prior study in the field(Wang H et al. Immunity. 2020 Jul 14;53(1):204-216.e10) .
2. Describe inclusion/exclusion criteria if samples or animals were excluded from the analysis. Were the criteria pre-established? (Give section/paragraph or page #)	No samples were excluded from the analysis.
3. If a method of randomization was used to determine how samples/animals were allocated to experimental groups and processed, describe it. (Give section/paragraph or page #)	It is unavalible to randomly assigned mice in this study due to the all experiments required specific genotypes.
For animal studies, include a statement about randomization even if no randomization was used.	Manuscript MATERIALS AND METHODS, page7

4. If the investigator was blinded to the group allocation during the experiment and/or when assessing the outcome, state the extent of blinding. (Give section/paragraph or page #)	For RNA-seq library preparetion and sequencing, investigators were blinded to experimental condition.
For animal studies, include a statement about blinding even if no blinding was done.	Blinding was not suitble in mouse studies, because treated models and specific genotypes had to be identified according to experimental designs.
5. For every figure, are statistical tests justified as appropriate?	Yes
Do the data meet the assumptions of the tests (e.g., normal distribution)?	Yes
Is there an estimate of variation within each group of data?	Yes
Is the variance similar between the groups that are being statistically compared? (Give section/paragraph or page #)	Yes

Reagents

Reported in section/paragraph or page #

6. Report the source of antibodies (vendor and catalog number)	Manuscript MATERRIALS AND METHODS, page8
7. Identify the source of cell lines and report if they were recently authenticated (e.g., by STR profiling) and tested for mycoplasma contamination	Cell lines are derived from ATCC.All cell lines were tested negative for mycoplasma cintamination.

Animal Models

Reported in section/paragraph or page #

8. Report species, strain, sex and age of animals	Manuscript MATRIALS AND METHODS, page7
9. For experiments involving live vertebrates, include a statement of compliance with ethical regulations and identify the committee(s) approving the experiments.	Manuscript MATERIALS AND METHODS,page7

10. We recommend consulting the ARRIVE guidelines (*PLoS Biol.* **8**(6), e1000412,2010) to ensure that other relevant aspects of animal studies are adequately reported.

Human subjects

Reported in section/paragraph or page #

11. Identify the committee(s) approving the study protocol.	Manuscript Ethics Approval and Consent to participate , page22
12. Include a statement confirming that informed consent was obtained from all subjects.	Manuscript Ethics Approval and Consent to participate ,page22
13. For publication of patient photos, include a statement confirming that consent to publish was obtained.	N/A
14. Report the clinical trial registration number (at ClinicalTrials.gov or equivalent).	N/A

15. For phase II and III randomized controlled trials, please refer to the CONSORT statement and submit the CONSORT checklist with your submission.

16. For tumor marker prognostic studies, we recommend that you follow the REMARK reporting guidelines.

Data deposition

Reported in section/paragraph or page #

17. Provide accession codes for deposited data. Data deposition in a public repository is mandatory for:<br>a. Protein, DNA and RNA sequences<br>b. Macromolecular structures<br>c. Crystallographic data for small molecules<br>d. Microarray data	RNA sequencing data of this study have been deposited in NCBI BioProject with accession number PRJNA860003

Deposition is strongly recommended for many other datasets for which structured public repositories exist; more details on our data policy are available in the Guide to Authors. We encourage the provision of other source data in supplementary information or in unstructured repositories such as Figshare and Dryad. We encourage publication of Data Descriptors (see Scientific Data) to maximize data reuse.

18. If computer code was used to generate results that are central to the paper's conclusions, include a statement in the Methods section under "**Code availability**" to indicate whether and how the code can be accessed. Include version information as necessary and any restrictions on availability.	N/A
